# Advances in Understanding and Managing Autoimmune Hepatitis: A Narrative Review

**DOI:** 10.7759/cureus.43973

**Published:** 2023-08-23

**Authors:** Vikas Yadav, Rabbia Irfan, Shamayel Safdar, Vyshnavidevi Sunkara, Chukwuyem Ekhator, Praful R Pendyala, Monika Devi, S M Iram Shahzed, Archana Das, Maryam Affaf, Sophia B Bellegarde, Riya Shrestha, Muhammad Arsal Naseem, Ahmed Al Khalifa

**Affiliations:** 1 Internal Medicine, Pandit Bhagwat Dayal Sharma Post Graduate Institute of Medical Sciences, Rohtak, IND; 2 Internal Medicine, Mayo Hospital, Lahore, PAK; 3 Medicine, Katuri Medical College and Hospital, Guntur, IND; 4 Neuro-Oncology, New York Institute of Technology, College of Osteopathic Medicine, Old Westbury, USA; 5 Neurology, Chalmeda Anand Rao Institute of Medical Sciences, Karimnagar, IND; 6 Medicine, Ziauddin University, Karachi, PAK; 7 Internal Medicine, South Brooklyn Health, Brooklyn, USA; 8 Internal Medicine, North East Medical College and Hospital, Sylhet, BGD; 9 Medicine, Khyber Medical University, Peshawar, PAK; 10 Pathology and Laboratory Medicine, American University of Antigua, St. John’s, ATG; 11 Medicine, Nepal Medical College and Teaching Hospital, Kathmandu, NPL; 12 Medicine, Mayo Hospital, Lahore, PAK; 13 Medical School, College of Medicine, Sulaiman Alrajhi University, Al Bukayriyah, SAU

**Keywords:** hepatobiliary, inflammation, autoimmune liver disease, liver disease, hepatitis, autoimmune

## Abstract

Autoimmune hepatitis (AIH) is a chronic liver disease characterized by immune-mediated destruction of hepatocytes, leading to inflammation and fibrosis. In recent years, significant advances have been made in understanding the pathogenesis, epidemiology, diagnosis, and treatment of AIH. This comprehensive narrative review aims to provide an up-to-date overview of these advances. The review begins by outlining the historical background of AIH, dating back to its initial recognition in the 1940s, and highlights the evolution of diagnostic criteria and classification based on autoantibody profiles. The epidemiology of AIH is explored, discussing its varying prevalence across different regions and the role of genetic predisposition, viral infections, and drug exposure as risk factors. Furthermore, the review delves into the pathogenesis of AIH, focusing on the dysregulated immune response, involvement of T cells, and potential contribution of the gut microbiome. Clinical presentation, diagnostic criteria, and liver biopsy as crucial tools for diagnosis are also discussed. Regarding management, the review provides an in-depth analysis of the standard first-line treatments involving glucocorticoids and azathioprine, as well as alternative therapies for non-responsive cases. Additionally, emerging second and third-line treatment options are examined. In conclusion, this narrative review highlights the complexity of AIH and underscores the importance of early diagnosis and individualized treatment approaches to improve patient outcomes. Further research and clinical trials are needed to optimize AIH management and ensure a better long-term prognosis for affected individuals.

## Introduction and background

Autoimmune hepatitis (AIH) is a chronic inflammatory liver disease caused by an autoimmune attack on hepatocytes [1]. AIH traces back to the 1940s when instances of chronic hepatitis emerged, characterized by heightened serum proteins with a higher occurrence in females [2]. In the 1950s, Swedish physician Waldenström documented cases of hepatitis showcasing significant increases in serum gamma globulins and menstrual irregularities. These cases exhibited improvement following treatment with adrenocorticotropic hormone [3]. Originally referred to as chronic active hepatitis, the autoimmune underpinning of the condition was hinted at by the presence of lupus erythematosus cells in affected individuals. This led to its initial identification as “lupoid hepatitis,” though this term was later supplanted by AIH, as it became apparent that lupus erythematosus and AIH were distinct entities [4]. Once considered uncommon, the prevalence of AIH has risen worldwide, likely due to lifestyle changes and advancements in diagnostic capabilities [5].

AIH can be categorized into three subtypes based on autoantibody patterns [6]. The most prevalent is AIH type 1 (AIH-1), linked to the presence of antinuclear antibodies (ANAs), smooth muscle autoantibodies (SMAs), and perinuclear anti-neutrophil cytoplasmic antibodies (p-ANCAs). AIH type 2 is less frequent and typically affects children, identified by the existence of anti-liver/kidney microsomal antibody type 1 (anti-LKM1), anti-LKM type 3 (anti-LKM3), and/or antibodies against liver cytosol type 1 antigen (anti-LC1). AIH type 3 is associated with antibodies against soluble liver antigen (SLA) or liver-pancreas antigen (LP).

While the exact triggers of AIH remain incompletely comprehended, it is thought to arise in genetically predisposed individuals, triggered by factors such as infections [7]. The progression of AIH can lead to liver scarring and cirrhosis, underscoring the significance of effective evaluation and management. Yet, there is a scarcity of clearly defined and universally accepted criteria for diagnosing and treating various variants of autoimmune liver conditions, including AIH. The existence of shared common features and genetic vulnerabilities among autoimmune liver illnesses can lead to overlapping diagnoses, culminating in treatment conundrums. Consequently, there is a pressing demand for widely endorsed guidelines addressing the diagnosis and treatment of these disorders.

## Review

Epidemiology and risk factors

The occurrence of AIH displays global variability, with most epidemiological investigations originating from Western countries and, more recently, Asia. Initial studies, conducted before the establishment of diagnostic criteria, noted an occurrence rate of 0.1 to 1.9 cases per 100,000 people in European countries and Japan [1]. However, recent analyses from Europe have revealed a higher frequency of the disease, with a range of 1.1 to 2.56 incidents and a prevalence of 17.3 to 18.3 per 100,000 individuals [8]. Modern research suggests a further increase in disease incidence. In the United Kingdom, a population-focused study in primary care reported an annual AIH occurrence of 1.94 per 100,000 individuals from 2002 to 2016, noting a greater frequency at higher latitudes [9]. In the United States, an investigation based on a substantial commercial database unveiled a notable AIH prevalence of 31.2 per 100,000 people of all age groups from 2014 to 2019 [10]. Additionally, a population-oriented study from New Zealand recorded an overall incidence of 1.93 per 100,000 between 2008 and 2016, revealing a significant rise in incidence over time [11].

AIH predominantly affects females, with a male-to-female ratio ranging from 1:4 to 1:6 [7,12]. Although the prior assumption was that AIH primarily influenced individuals aged 40 to 60, recent exploration has demonstrated that older patients over the age of 70 can also develop AIH, often with more advanced stages at diagnosis [13]. The precise cause of the disease remains incompletely understood; however, it appears to entail genetic susceptibility, environmental elements, and epigenetic changes. Furthermore, emerging evidence proposes a link between AIH and the gut microbiome [14].

Viral infections have also been reported as risk factors for AIH, highlighting the pathogenetic process of molecular mimicry. This phenomenon arises when the immune response aimed at pathogens is redirected toward structurally similar self-antigens, potentially inciting AIH. Specifically, AIH-2 has been associated with shared amino acid sequences between the autoantigen CYP2D6 and proteins of the hepatitis C virus and other herpesviruses [1,15]. Exposure to drugs also represents a risk, as certain medications and herbal supplements can lead to drug-induced liver ailments resembling AIH, particularly in predisposed individuals. Noteworthy medications linked to this risk include nitrofurantoin, minocycline, anti-tumor necrosis factor-alpha (TNF-α) drugs, and statins [16].

Genetic predisposition assumes a substantial role in AIH susceptibility, particularly through polymorphisms in human leukocyte antigen (HLA) alleles. HLA-DRB1 alleles have been associated with AIH-1 vulnerability, and specific HLA types are more prevalent in particular populations, such as DRB10405 in Japan and Argentina and DRB10404 in Mexico [17-19]. Moreover, AIH-2 is linked to DRB107 and DRB103, while pediatric AIH-1 is linked to DRB103 in northern Europe [20]. Pediatric AIH patients might exhibit isolated partial deficiency of the HLA class III complement component C4, and AIH-2 can be part of the autoimmune polyendocrinopathy-candidiasis-ectodermal dystrophy syndrome [21]. Non-HLA genetic polymorphisms have also been associated with AIH susceptibility, albeit with less pronounced effects [22].

Pathogenesis

AIH displays a distinctive feature of intense inflammation within the liver, marked by the infiltration of lymphocytes, macrophages, and plasma cells [23]. Although AIH is linked to circulating autoantibodies and the infiltration of plasma cells into the liver, it is primarily recognized as a T-cell-driven disorder, with B-cell activation contingent on T-cell involvement. The influential role of T cells becomes apparent through the susceptibility conferred by HLA class II polymorphisms [24].

The onset of AIH pathogenesis is believed to originate with the introduction of self-antigenic peptides, such as CYP2D6 and FTCD in AIH-2 and the human SepSecS-tRNASec complex in AIH-1, to naive CD4 T-helper (Th0) lymphocytes [8]. This presentation occurs within antigen-presenting cells, either in the regional lymph nodes or directly in the liver. Following activation, Th0 cells differentiate into Th1 or Th2 cells depending on the presence of interleukin (IL)-12 or IL-4, respectively, as well as the nature of the antigen. Th1 cells release IL-2 and interferon-gamma (IFN-γ), leading to the activation of cytotoxic T lymphocytes and heightened expression of class I HLA molecules on hepatocytes, which can induce damage to liver cells [25]. IFN-γ also induces the expression of class II HLA molecules on hepatocytes, further amplifying T-cell activation [1]. Conversely, Th2 cells foster B-cell activation and the production of autoantibodies [26]. Although the role of Th17 cells, which generate inflammatory cytokines, has been explored, its exact contribution to AIH remains somewhat unclear [27].

Regulatory T cells (Tregs) play a vital role in preserving immune tolerance and preventing autoimmune responses. In AIH, Tregs exhibit deficiencies in both quantity and function, enabling a dysregulated immune reaction against liver antigens [28]. Reestablishing adequate Treg levels and function can hold potential as a treatment strategy. The gut microbiome has also surfaced as a possible contributor to AIH pathogenesis. Disruptions in gut bacteria composition (dysbiosis) have been detected in AIH, and the connection between the gut and liver might contribute to the emergence of autoimmunity centered on the liver [29].

Persistent liver damage in AIH leads to the activation of liver cells and inflammatory agents, prompting inflammation and oxidative stress. This cascade results in heightened collagen synthesis and an excess accumulation of extracellular matrix, which, in turn, leads to fibrosis. Central to this fibrogenic response are hepatic stellate cells and myofibroblasts [30]. The fibrogenic cytokines and chemokines produced during the inflammatory process trigger the transformation of hepatic stellate cells into myofibroblasts, driving the production of extracellular matrix and the progression of fibrosis. Moreover, the apoptosis of liver cells and the generation of apoptotic bodies contribute to the advancement of fibrosis in AIH [31]. The instigation of oxidative stress, stemming from an imbalance between the production and elimination of reactive oxygen species (ROS), further exacerbates liver impairment and fibrosis [32].

Clinical presentation

The clinical manifestation of AIH among adults exhibits a wide spectrum, spanning from asymptomatic instances to acute liver failure. Around two-thirds of adult patients encounter a gradual initiation, presenting either without symptoms or with non-specific symptoms such as fatigue, malaise, loss of appetite, joint pain, and weight loss [33]. In young females, a typical presenting sign is amenorrhea, and AIH can arise during pregnancy or shortly thereafter. Irrespective of ethnicity, gender, or age, AIH should be considered as a possible diagnosis for individuals displaying elevated liver enzyme levels [34]. While heightened IgG levels are indicative of AIH, roughly 10% of patients might exhibit normal IgG levels at the time of diagnosis. Approximately one-third of patients present with acute disease, either as an acute exacerbation of chronic AIH or as primary acute AIH without indications of chronic liver ailment [7,35]. In cases of acute presentation, autoimmune serology tests might yield negative results, and IgG levels could be within the normal range, making diagnosis intricate. Regrettably, one-third of patients are already in a cirrhotic state at the time of diagnosis [36]. The severity of cirrhosis upon diagnosis might carry prognostic implications, as patients presenting with severe ascites tend to have lower transplant-free survival compared to those with compensated cirrhosis. Additionally, AIH patients demonstrate an increased occurrence of autoimmune disorders beyond the liver, with autoimmune thyroid disease being the most prevalent.

Pediatric instances of AIH can be categorized into the following two primary types: AIH-1, which typically emerges during adolescence, and AIH-2, which affects younger children, including infants [37]. The clinical demonstration in children mirrors that of adults, although acute onsets are more common, accounting for up to two-thirds of cases. AIH-2 is more prone to exhibit a fulminant presentation. Children diagnosed with AIH-2 often show concurrent autoimmune skin manifestations, such as vitiligo, alopecia, cutaneous vasculitis, and urticarial [38]. Roughly half of pediatric AIH patients have cirrhosis at the point of diagnosis [39].

Diagnosis

The diagnosis of AIH entails the assessment of diverse clinical, serological, biochemical, and histological attributes. Over the years, several diagnostic criteria have been proposed to aid in identifying AIH; however, some of the earlier scoring systems were intricate and of questionable utility [40,41]. In 2008, the International Autoimmune Hepatitis Group streamlined the diagnostic criteria into the following four parameters: liver histology, titers of autoantibodies (including ANA, SMA, anti-LKM1, anti-LKM3, and anti-LC1 autoantibodies), serum levels of γ-globulin or IgG, and absence of viral hepatitis [42]. This simplified scoring system exhibited high specificity and sensitivity for confirming probable and definite AIH. Nevertheless, it was noted to be less sensitive in ruling out AIH in patients with concurrent liver disorders, underscoring the significance of liver biopsy in select cases [1,7]. In instances of severe acute AIH, the simplified scoring system might yield lower diagnostic accuracy as patients can exhibit normal IgG levels and negative autoantibodies [43].

Although diagnostic scoring systems have been devised for adults, they might not apply to pediatric AIH cases. The European Society of Pediatric Gastroenterology, Hepatology, and Nutrition has established a pediatric diagnostic scoring system that takes into account distinct cutoff values and additional tests, such as adjusted autoantibody titers and the measurement of peripheral anti-nuclear neutrophil antibodies [37]. In children diagnosed with AIH, guidelines advocate for a cholangiogram to rule out autoimmune sclerosing cholangitis, which can exhibit similar clinical attributes. However, cholangiography is not included in the guidelines provided by the American Association for the Study of Liver Diseases (AASLD) [44].

Detecting autoantibodies is pivotal for both diagnosing and classifying AIH. Individuals with AIH-1 test positive for ANA and/or SMA, whereas AIH-2 patients show positivity for anti-LKM1 or, in rare cases, anti-LKM3, and/or anti-LC1 autoantibodies [13,45]. Once regarded as a distinct category, the presence of anti-SLA/LP autoantibodies was later found to share similar characteristics with AIH-1 patients [46]. However, the presence of anti-SLA/LP autoantibodies proves particularly beneficial in patients who lack conventional antibodies.

Liver biopsy remains a fundamental aspect of AIH diagnosis, offering a definitive identification and allowing the assessment of histological attributes. The simplified diagnostic criteria for AIH mandate the presence of the following three histological characteristics: interface hepatitis, emperipolesis (the occurrence of a plasma cell or lymphocyte within hepatocytes), and rosettes (small clusters of hepatocytes arranged around a central lumen) [42]. Recent studies have proposed that while emperipolesis and rosettes are not exclusively indicative of AIH, they might serve as surrogate markers for disease severity [47]. Patients who are ineligible for liver biopsy could gain from non-invasive techniques to assess liver fibrosis, such as transient elastography (TE). In addition, the innovative FibroMeter vibration-controlled TE (FMVCTE) technique combines FM values and LSMs to identify severe fibrosis in AIH patients [48].

Treatment

The primary objective in treating AIH is to attain biochemical remission, which entails achieving normal serum transaminase and IgG levels. In children, remission also involves negative or low-titer autoantibodies, as certain autoantibodies in this age group correlate with disease activity [44]. The attainment of biochemical remission holds significance as it corresponds with improved histological activity and halts disease progression. Notably, AIH-related symptoms abate upon the achievement of biochemical remission.

The conventional treatment for AIH comprises two core components, namely, glucocorticoids (steroids) and azathioprine. Glucocorticoids play a pivotal role in AIH treatment for both children and adults, efficiently driving biochemical remission in the majority of patients. Their mode of action involves binding to the glucocorticoid receptor, repressing pro-inflammatory genes, and activating anti-inflammatory genes [49]. They also stimulate the proliferation of Tregs [50]. These steroids, especially predniso(lo)ne, constitute the preferred choice in AIH treatment. The initial dosage of predniso(lo)ne is contingent on the severity of the disease. The European Association for the Study of the Liver guidelines propose a dose ranging from 0.5 to 1 mg/kg/day in adults [34]. AASLD guidelines recommend initiating 60 mg/day in cases of acute severe AIH and 20-40 mg/day in other instances. In children, the recommended starting dose is 2 mg/kg/day (up to a maximum of 60 mg/day) [44]. Predniso(lo)ne is gradually tapered weekly, and a rapid reduction in serum transaminase levels within the initial eight weeks of treatment predicts transaminase normalization at later points. Azathioprine, acting as the primary steroid-sparing agent in AIH, is typically introduced a few weeks into steroid treatment. It inhibits purine synthesis, leading to the demise of rapidly dividing cells, including lymphocytes. Azathioprine should be initiated at low doses, followed by gradual increments to the maintenance dose, considering potential hepatotoxicity and side effects. Monitoring blood cell counts is vital due to its potential myelotoxicity [51].

The second-line treatment for AIH comes into play when patients either cannot tolerate or show inadequate response to standard first-line therapies, such as predniso(lo)ne and azathioprine. Multiple options are available for such scenarios. 6-Mercaptopurine (6-MP) can serve as an alternative for patients intolerant to azathioprine [52]. For azathioprine-intolerant AIH patients, another possibility is 6-thioguanine, which is enzymatically converted to its active metabolite, 6-thioguanine nucleotide, bypassing the step that leads to the formation of the toxic metabolite 6-methylmercaptopurine [53]. However, its use in AIH is less established, and high doses have been linked to an elevated risk of non-cirrhotic portal hypertension. Mycophenolate mofetil (MMF) curbs purine synthesis in B and T lymphocytes. It is employed off-label in AIH patients who cannot tolerate azathioprine and 6-MP or those who show an unsatisfactory response to standard therapy [34,44]. MMF has shown to be more effective in the latter group and is generally better tolerated than azathioprine. Yet, its main drawback is its teratogenic potential, which poses concerns for women of childbearing age [44].

The third-line treatment is reserved for approximately 10-20% of AIH patients who prove challenging to treat and are often managed in specialized centers. For such cases, a combination of immunosuppressive drugs is often needed, although the evidence is primarily based on retrospective series and experiences from single centers rather than randomized controlled trials. Calcineurin inhibitors (CNIs), such as cyclosporin A and tacrolimus, have been employed as rescue therapies in both adults and children with AIH [54,55]. While CNIs have demonstrated effectiveness in specific instances, they are associated with significant long-term toxicity, including neurotoxicity, renal injury, hypertension, diabetes, hyperlipidemia, and secondary malignancies. mTOR inhibitors regulate the proliferation and survival of activated lymphocytes [56]. Few documented cases exist of AIH patients unresponsive to standard drugs treated with mTOR inhibitors, yet these are associated with significant side effects such as hyperlipidemia, mouth and leg ulcers, thrombocytopenia, proteinuria, rash, and heightened susceptibility to infections [56].

B-cell depletion therapies, including rituximab (anti-CD20 antibody) and ianalumab (anti-B-cell activating factor receptor), have demonstrated positive effects in AIH patients who fail to respond to standard therapies [57,58]. Drugs that inhibit TNF-α have also been employed in various autoimmune and autoinflammatory conditions. While infliximab has shown effectiveness in normalizing serum transaminase levels in certain adult AIH patients, caution is warranted due to potential complications, including drug-induced liver injury resembling AIH [59].

Managing acute severe AIH or fulminant liver failure remains a challenge, often necessitating prompt consideration of liver transplantation in severe cases [44]. Careful management of treatment withdrawal is essential, and a liver biopsy is advisable before discontinuing treatment [34]. It is vital to note that many third-line treatments mentioned above are grounded in limited evidence and necessitate further research, preferably through randomized controlled trials, to establish their safety and efficacy in AIH management.

## Conclusions

This comprehensive narrative review highlights the significant advances in our understanding and management of AIH. Over the years, AIH has transformed from being considered a rare disorder to an increasingly prevalent and complex autoimmune liver disease. Advancements in diagnostic criteria, serological markers, and liver biopsy techniques have facilitated accurate and timely diagnosis, enabling early intervention and improved patient outcomes. Standard treatment protocols involving glucocorticoids and azathioprine have shown efficacy in achieving biochemical remission in the majority of patients, but challenges remain in managing non-responsive cases. Furthermore, the review sheds light on emerging therapeutic strategies, such as B-cell depletion therapies and novel immunomodulatory agents, which hold promise for refractory AIH cases. However, more research is needed to establish their long-term safety and efficacy. Overall, this narrative review emphasizes the importance of a multidisciplinary approach, incorporating clinical, serological, histological, and genetic factors, to optimize the management of AIH. The evolving landscape of AIH research underscores the need for ongoing collaboration and research to further enhance our understanding and refine therapeutic approaches, ultimately improving the quality of life for AIH patients worldwide.
